# ATRX, IDH1-R132H and Ki-67 immunohistochemistry as a classification scheme for astrocytic tumors

**DOI:** 10.18632/oncoscience.317

**Published:** 2016-09-06

**Authors:** Jinquan Cai, Chuanbao Zhang, Wei Zhang, Guangzhi Wang, Kun Yao, Zhiliang Wang, Guanzhang Li, Zenghui Qian, Yongli Li, Tao Jiang, Chuanlu Jiang

**Affiliations:** ^1^ Department of Neurosurgery, The Second Affiliated Hospital of Harbin Medical University, NanGang District, Harbin, Heilongjiang Province 150001, China; ^2^ Beijing Neurosurgical Institute, Capital Medical University, Dongcheng District, Beijing 100050, China; ^3^ Department of Neurosurgery, Beijing Tiantan Hospital, Capital Medical University, Dongcheng District, Beijing 100050, China; ^4^ Beijing Institute for Brain Disorders Brain Tumor Center, Dongcheng District, Beijing 100050, China; ^5^ China National Clinical Research Center for Neurological Diseases, Dongcheng District, Beijing 100050, China; ^6^ Chinese Glioma Cooperative Group (CGCG), Dongcheng District, Beijing 100050, China; ^7^ Department of Pathology, Sanbo Brain Hospital, Capital Medical University, Haidian District, Beijing 100093, China

**Keywords:** ATRX, IDH-R132H, Ki-67, astrocytic tumors, progression

## Abstract

Recurrence and progression to higher grade lesions are key biological events and characteristic behaviors in the evolution process of glioma. Malignant astrocytic tumors such as glioblastoma (GBM) are the most lethal intracranial tumors. However, the clinical practicability and significance of molecular parameters for the diagnostic and prognostic prediction of astrocytic tumors is still limited. In this study, we detected ATRX, IDH1-R132H and Ki-67 by immunohistochemistry and observed the association of IDH1-R132H with ATRX and Ki-67 expression. There was a strong association between ATRX loss and IDH1-R132H (p<0.0001). However, Ki-67 high expression restricted in the tumors with IDH1-R132H negative (p=0.0129). Patients with IDH1-R132H positive or ATRX loss astrocytic tumors had a longer progressive- free survival (p<0.0001, p=0.0044, respectively). High Ki-67 expression was associated with shorter PFS in patients with astrocytic tumors (p=0.002). Then we characterized three prognostic subgroups of astrocytic tumors (referred to as A1, A2 and A3). The new model demonstrated a remarkable separation of the progression interval in the three molecular subgroups and the distribution of patients’ age in the A1-A2-A3 model was also significant different. This model will aid predicting the overall survival and progressive time of astrocytic tumors’ patients.

## INTRODUCTION

Astrocytic tumors are the most common group of human gliomas with inherent tendency for recurrence and malignant progression [1]. Malignant astrocytic tumors such as glioblastoma (GBM) are the most lethal intracranial tumors [2]. The clinical outcome of patients with astrocytic tumors depends on several factors, most notably age at diagnosis, clinical status as measured by the Karnofsky score, and tumor resection extent, as well as the histological classification, tumor grade and key molecular genetic alteration [3]. Despite the treatment of neurosurgical resection, radiotherapy and chemotherapy, most astrocytic tumors still grow continuously and tend to progress to a higher grade, leading to neurological disability and ultimately to death [4]. During recent years, large-scale research efforts – spearheaded by The Cancer Genome Atlas (TCGA) have made rapid advances in understanding glioma genetics. Isocitrate dehydrogenase (IDH) mutations were detected in approximately 80 % of diffuse and anaplastic astrocytomas as well as secondary GBMs [5]. The oncogenic IDH1 mutations mainly represented a change of guanine to adenine at position 395 (G395A), leading to the replacement of arginine by histidine at codon 132 (IDH1-R132H) at the enzymatic active site [5, 6]. Patients with IDH1-R132H positive astrocytic tumors had a better outcome than those with IDH1-R132H negative astrocytic tumors. Recently, several teams demonstrated that IDH mutations and ATRX status, combined with other classical biomarkers, refined the molecular classification of adult gliomas, providing a prognostic tool for clinicians [7–11]. These studies supported the development of a new molecular classification of IDH1-R132H and loss of ATRX, in that the clinical characteristics and prognosis of patients with grade II/III glioma and GBM are not accurately reflected by histological classifications [8, 9, 11–13]. The “Haarlem Consensus Guidelines for Nervous System Tumor Classification” [14] suggest that some entities will require molecular information to provide an “integrated” diagnosis, which is based on several layers comprising (i) the integrated diagnosis as top layer, followed by (ii) histological classification, (iii) WHO grade, and (iv) molecular information [14]. However, the clinical practicability and significance of molecular parameters for the diagnostic and prognostic prediction of astrocytic tumors is still limited. In this study, we detected ATRX, IDH1-R132H and Ki-67 by immunohistochemistry and characterized three prognostic subgroups of astrocytic tumors (referred to as A1, A2 and A3).

## RESULTS

### Detection of ATRX, IDH1-R132H and Ki-67 in astrocytic tumors by immunohistochemistry

**Figure 1:**
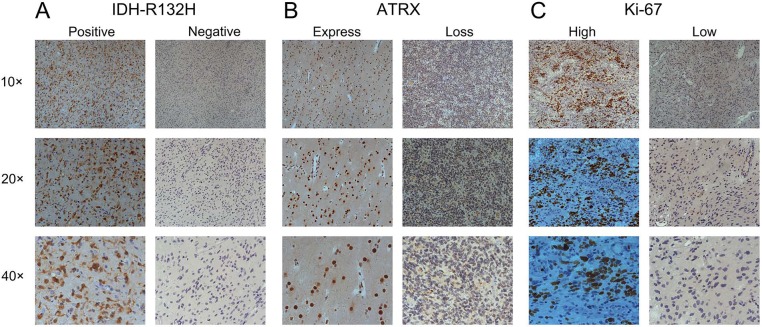
Detection of ATRX, IDH1-R132H and Ki-67 in astrocytic tumors by immunohistochemistry **A.** IDH1-R132H positive or negative (10 ×, 20 ×, 40×). **B.** ATRX nuclear protein expression or loss (10 ×, 20 ×, 40×). **C.** Ki-67 high or low expression (10 ×, 20 ×, 40×).

In our dataset, ATRX nuclear protein, IDH1-R132H and Ki-67 was detected by immunohistochemistry. IDH1- R132H dominated in diffuse astroctytoma (29/50, 58%) and anaplastic astrocytoma (5/9, 55.6%) compared with in primary GBMs (9/58, 15.5%) (Figure 1A; Table 1, p<0.0001, Chi-Square test). The frequency of ATRX loss was higher in As (41/50, 82%) and AAs (7/9, 77.8%) than that in pGBMs (7/58, 12.1%) (Figure 1B; Table 1, p<0.0001, Chi-Square test). Primary GBMs (50/58, 86.2%) expressed higher Ki-67 protein than diffuse and anaplastic astrocytoma (1/58, 1.7%) (Figure 1C; Table 1, p<0.0001, Chi-Square test).

**Table 1:** Clinicopathological characteristics of the patients in the cohort

Variables		A (N=50)	AA (N=9)	GBM (N=58)	p value
**Median age (year)**		38	39	44	0.0566
**Age**	≤ 42 years old > 42 years old	32 18	6 3	23 35	0.0273
**Gender**	Male Female	28 22	5 4	33 25	0.9942
**IDH1-R132H**	Positive Negative	29 21	5 4	9 49	< 0.0001
**ATRX-loss**	Yes No	41 9	7 2	7 51	< 0.0001
**Ki-67 Expression**	High Low	1 49	0 9	50 8	< 0.0001

### Association of IDH1-R132H with ATRX and Ki-67 expression

**Figure 2:**
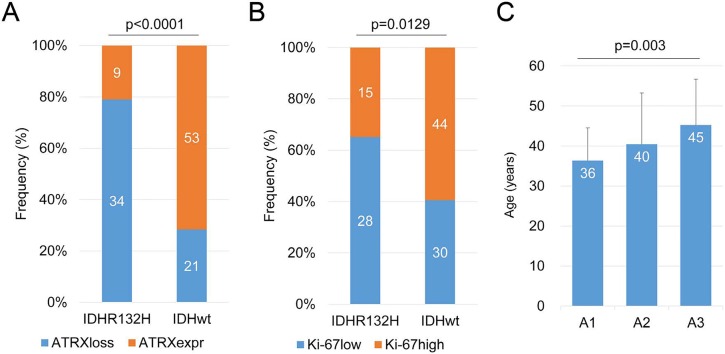
Correlation of ATRX and Ki-67 protein expression with IDH1-R132H status **A.** There was a strong association between ATRX loss and IDH1-R132H (p<0.0001). **B.** However, Ki-67 high expression restricted in the tumors with IDH1-R132H negative (p=0.0129). **C.** The distribution of patients’ age in the A1-A2-A3 model was significant different (p=0.003).

In accord with previous reports, among the 41 tumor samples with IDH1-R132H positive, 34 lacked ATRX nuclear protein, while 53 samples of 74 astrocytic tumors with IDH1-R132H negative expressed ATRX, indicating a strong association between ATRX loss and IDH1-R132H (Figure 2A; p<0.0001, Chi-Square test). In addition, the Ki-67 high expression restricted in the tumors with IDH1- R132H negative, indicating a group astrocytic tumors with high cell proliferative capacity (Figure 2B; p=0.0129).

### Prognostic value of ATRX, IDH1-R132H and Ki- 67 for patients with astrocytic tumors

**Figure 3:**
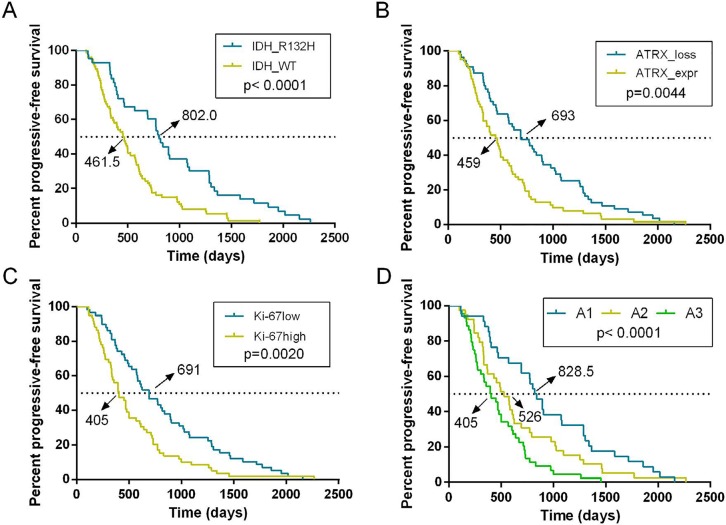
Kaplan-Meier estimates of survival for astrocytic tumor patients **A.** Patients with IDH1-R132H positive astrocytic tumors had a longer progressive-free survival than those with IDH1-R132H negative astrocytic tumors (p<0.0001). **B.** ATRX loss was also a favorable prognostic factor in patients with astrocytic tumors (p=0.0044). **C.** High Ki-67 expression was associated with shorter PFS in patients with astrocytic tumors (p=0.002). **D.** Survival analysis of the new classification revealed a remarkable separation of the clinical course in the three molecular subgroups (p<0.0001).

Then we evaluated the value of ATRX, IDH1- R132H and Ki-67 for predicting the progression of astrocytic tumors. We observed that patients with IDH1-R132H positive astrocytic tumors had a longer progressive-free survival (PFS) than those with IDH1- R132H negative astrocytic tumors (Figure 3A; Median PFS of IDH1-R132H positive=802 days, Median PFS of IDH1-R132H negative=461.5 days; p<0.0001). ATRX loss was also a favorable prognostic factor in patients with astrocytic tumors (Figure 3B; Median PFS of ATRX loss=693 days, Median PFS of ATRX expression=459 days; p=0.0044). High Ki-67 expression was associated with shorter PFS in patients with astrocytic tumors (Figure 3C; Median PFS of high Ki-67 expression=405 days, Median PFS of low Ki-67 expression =459 days; p=0.002).

### Classification of astrocytic tumors defined by ATRX, IDH1-R132H and Ki-67

**Figure 4:**
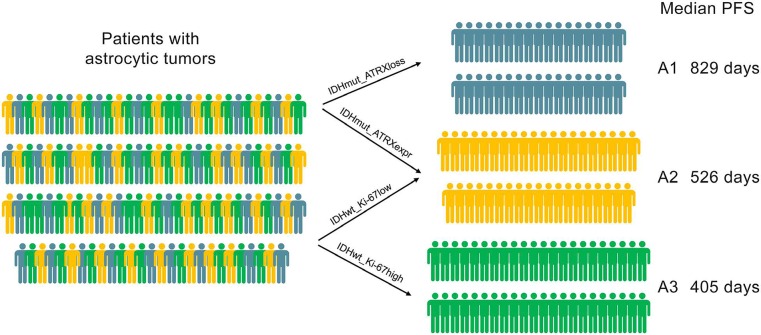
A1-A2-A3 Model for classification of astrocytic tumors based on ATRX, Ki-67 and IDH1-R132H status IDH1-R132H positive tumors with ATRX loss were defined as A1 (median PFS=829 days), IDH1-R132H negative tumors with high Ki-67 expression as A3 (median PFS=526 days), and IDH1-R132H positive tumors with ATRX expression and IDH1-R132H negative tumors with low Ki-67 expression were termed as A2 (median PFS=405 days).

Based on the above results and previous research, we incorporated IDH1-R132H, ATRX and Ki-67 status detected by IHC into A1-A2-A3 model. We classified astrocytic tumors into IDH1-R132H positive and IDH1- R132H negative tumors and then defined IDH1-R132H positive and ATRX loss tumors as A1, IDH1-R132H negative tumors with high Ki-67 expression as A3, and grouped IDH1-R132H positive with ATRX expression and IDH1-R132H negative tumors with low Ki-67 expression into A2. We observed that the distribution of patients’ age in the A1-A2-A3 model was significant different (Figure 2C, p=0.003). As showed in Figure 3D, survival analysis of the new classification also demonstrated a remarkable separation of the clinical course in the three molecular subgroups (log-rank test, p<0.0001). The A1 subgroup was correlated with a better clinical outcome (Figure 4, Median PFS = 828.5 days). In contrast, the A3 subgroup was associated with a poorer clinical outcome (Median PFS = 405 days). Correlation of the A2 subgroup with respect to clinical outcome fell between the A1 and A3 subgroups (Median PFS = 526 days). To study the influence of the three molecular markers (IDH1, ATRX and Ki-67) we used in our classification schema, multivariate Cox regression analyses were used for the adjustment of these factors (Table 2). We confirmed that IDH1-R132H status and Ki-67 expression were the independent prognostic factors in this cohort and the new classification scheme was dependent on these three factors to predict survival value. Furthermore, upon incorporation of only our classification and the WHO grading scheme (Table 2), the prognostic value of our classification was still significant, independent of the WHO grades, and served as an addition to the latter.

**Table 2:** Cox regression analysis for the progression-free survival of patients with astrocytic Tumors

Variable	Univariate Cox Model			Multivariate Cox Model		
	HR	95%CI	p value	HR	95%CI	p value
**COX model of Classification, IDH, ATRX and Ki-67**						
Age ≤ 42 vs.>42	1.366	0.944-1.976	0.098			
Male vs. Female	0.924	0.635-1.346	0.682			
IDH1-R132H positive vs. negative	0.432	0.288-0.648	4.97E-05	0.218	0.067-0.71	0.012
ATRX loss vs. expression	0.586	0.404-0.851	5.00E-03	0.941	0.559-1.585	0.82
Ki-67 expression low vs. high	1.787	1.231-2.596	0.002	2,738	1.139-6.581	0.024
Classification	1.672	1.316-2.126	2.63E-05	0.5	0.188-1.330	0.165
**COX model of Classification and Grade**						
Classification	1.672	1.316-2.126	2.63E-05	1.512	1.062-2.153	0.022
WHO grade II/III vs. IV	1.449	1.187-1.770	2.70E-04	1.121	0.835-1.504	0.447

## DISCUSSION

Over the past decade, insights into the molecular pathology of gliomas have significantly improved both our biological understanding of neoplasms as well as our abilities to diagnose tumors and estimate their prognosis and likelihood of response to specific therapies [15]. In the present study, we detected the IDH1-R132H, ATRX and Ki-67 expression using immunohistochemistry, promoting the molecular neuropathology research to the clinical translational practice.

Nowadays, mutations in *IDH1* are commonly established as a hallmark molecular feature of grade II/III gliomas and secondary GBM which have predominant ocalization in the frontal and temporal lobes [15]. IDH1–R132H (G395A) is the most common mutation (∼90%), followed at a distance by IDH1– R132S (C394A), IDH1–R132C (C394T), DH1–R132G (C394G), IDH1–R132L (G395T) and IDH1–R132V (C394G G395T) (0.5–5%) [12]. Thus, IDH1-R132H can be used for the diagnosis between grade II/III gliomas, secondary GBM and primary GBM [16]. Mutations in the *IDH* genes are thought to cause glioma-CpG island methylator phenotype (G-CIMP) within the proneural GBM subgroup [17]. *IDH* mutations seem to require cooperating mutations in ATRX, and they are less frequently detected in primary GBMs [5]. Mutations of ATRX inactivated the gene product and caused a lack of ATRX immunolabeling [18]. ATRX loss occurs almost exclusively in IDH mutant astrocytic tumors, and ATRX loss and 1p/19q codeletion are largely mutually exclusive [19]. ATRX loss is characteristic in the refinement of the diagnosis of IDH mutant astrocytomas. Assessment of ATRX loss by immunohistochemical staining captures the majority of mutations, indicating that the use of immunohistochemical testing in routine neuropathology diagnostics gives a reasonable sensitivity [20].

In addition, our result showed that higher Ki- 67 expression mostly dominated in the IDH1-R132H negative cluster. Previously, our research delineated that IDH-wt/TERTp-mut gliomas expressed higher Ki- 67 protein and showed the evidence of cell proliferation. “Classical” gene expression was mostly restricted to the IDH-wt/TERTp-mut gliomas with the poorest survival. Now, we used negative IDH1-R132H combined with higher Ki-67 expression to define the cluster similar to the IDH-wt/TERTp-mut gliomas. In contrast with *IDH* mutations and ATRX loss being widely considered as key aberrations in the early stage of astrocytic tumors, higher Ki-67 expression may be the final event in the progression of these tumors. We speculated that IDH1- R132H accompanied by ATRX or Ki-67 may represent a distinct biological process during the development of astrocytic tumors from the original tumor cells.

Based on the above results and previous research, we incorporated IDH1-R132H, ATRX and Ki-67 status detected by IHC into A1-A2-A3 model. The new classification also demonstrated a remarkable separation of the progression interval in the three molecular subgroups and the distribution of patients’ age in the A1-A2-A3 model was also significant different. This model will aid predicting the overall survival and progressive time of astrocytic tumors’ patients.

## MATERIALS AND METHODS

### Patients enrollment

As a part of the Chinese Glioma Genome Atlas (CGGA) project (http://www.cgga.org.cn/portal.php), we consented patients who underwent surgical resection for malignant gliomas at the Glioma Treatment Center of Beijing Tiantan Hospital from January 2008 through March 2015. The study was approved by the ethics committee in both hospitals and written informed consent was obtained from each patient. All of data and samples were collected under the IRB of Beijing Tiantan Hospital. The criteria of enrollment include: age more than 18 years-old, histologically confirmed astrocytic tumors, relapse detected by MRI and patient's consent. 117 samples came into the cohort, containing astrocytoma (A, grade II), anaplastic astrocytoma (AA, grade III) and primary glioblastoma (GBM, grade IV). The histological diagnoses were confirmed by two neuropathologists according to the 2007 World Health Organization (WHO) classification guidelines. Specimens were collected after definitive diagnosis and stored as paraffin embedded blocks for subsequent molecular characterization. The collected specimens were verified by our pathologists to harbor >80% viable tumor tissue. For each enrolled patient, patients’ progression-free survival data were recorded when the relapse occurred.

### Immunohistochemistry for IDH1-R132H, ATRX and Ki-67

Immunostaining was performed according to the manufacturer's protocol. In brief, formalin-fixed, paraffinembedded tissue sections cut to four micrometer were dried at 80°C for 15 min and dewaxed in xylene, rinsed in graded ethanol, and rehydrated in double-distilled water. The sections were then treated with 3% H2O2 for 5 min at room temperature (RT) to block endogenous peroxidase activity. For antigen retrieval, slides were pretreated by steaming in sodium citrate buffer (10 mM sodium citrate, pH 6.0) for 15 min at 100°C. After washing with phosphate-buffered saline for 3 min, the sections were immunostained with an anti-human IDH1-R132H antibody (at 1:60 dilution, H09, Dianova, Hamburg, Germany) or an anti-human ATRX antibody (at 1:800 dilution, ab97508, Abcam) or an anti-human Ki-67 protein antibody (Santa Cruz Biotechnology, Santa Cruz, CA), and incubated at 4°C over night. After washed by 3 changes of PBS buffer, the tissues were covered by anti-mouse/rabbit polymer HRP-label for 30min at RT. Staining reaction was performed through covering tissue by prepared DAB chromogen solution, and incubating approximately for 10 min to allow for proper brown color development. Each slide was individually reviewed and scored by two experienced neuropathologists.

Standard of IDH1-R312H staining: (1) a strong cytoplasmic immunoreaction product was scored positive; (2) a weak diffuse staining and staining of macrophages were not scored positive [21, 22].

Standard of ATRX staining according to German Cancer Research Center (DKFZ): nuclear ATRX loss was scored as specific if tumor cell nuclei were unstained while nuclei of non-neoplastic cells such as endothelia, microglia, lymphocytes and reactive astrocytes were strongly positive [12]. Staining was scored using a two-grade scale, with 0=no or <10% occurrence of staining, 1 = >10% of cells positively stained. Score 1 was defined as high Ki-67 expression [5].

Staining was scored using a two-grade scale, with 0=no or <10% occurrence of staining, 1 = >10% of cells positively stained. Score 1 was defined as high Ki-67 expression [5].

### Statistical analysis

Receiver operating characteristic (ROC) curves were constructed to determine the discriminatory capacity of IDH1-R132H and (or) ATRX loss for diagnosis. Kaplan- Meier analysis was performed to estimate the survival time of different subgroups and a log-rank test was used to test prognostic differences. Comparisons of binary and categorical patient characteristics between subgroups were performed by the use of the Fisher's exact test All statistical computations were performed with the statistical software environment R version 3.2.0 (http://www.r-project.org/), GraphPad Prism Version 6.01 or Microsoft Excel 2013. P value < 0.05 was considered statistically significant.
